# *In vitro* extracellular replication of *Wolbachia* endobacteria

**DOI:** 10.3389/fmicb.2024.1405287

**Published:** 2024-07-18

**Authors:** Lara Vanessa Behrmann, Kirstin Meier, Jennifer Vollmer, Chukwuebuka Chibuzo Chiedu, Andrea Schiefer, Achim Hoerauf, Kenneth Pfarr

**Affiliations:** ^1^Institute for Medical Microbiology, Immunology and Parasitology, University Hospital Bonn, Bonn, Germany; ^2^German Center for Infection Research (DZIF), Partner Site Bonn-Cologne, Bonn, Germany

**Keywords:** *Wolbachia*, cell-free, endosymbionts, intracellular bacteria, *in vitro* culture, filariasis, vector control

## Abstract

Obligate intracellular endobacteria of the genus *Wolbachia* are widespread in arthropods and several filarial nematodes. Control programs for vector-borne diseases (dengue, Zika, malaria) and anti-filarial therapy with antibiotics are based on this important endosymbiont. Investigating *Wolbachia*, however, is impeded by the need for host cells. In this study, the requirements for *Wolbachia w*AlbB growth in a host cell-free *in vitro* culture system were characterized via qPCRs. A cell lysate fraction from *Aedes albopictus* C6/36 insect cells containing cell membranes and medium with fetal bovine serum were identified as requisite for cell-free replication of *Wolbachia*. Supplementation with the membrane fraction of insect cell lysate increased extracellular *Wolbachia* replication by 4.2-fold. Replication rates in the insect cell-free culture were lower compared to *Wolbachia* grown inside insect cells. However, the endobacteria were able to replicate for up to 12 days and to infect uninfected C6/36 cells. Cell-free *Wolbachia* treated with the lipid II biosynthesis inhibitor fosfomycin had an enlarged phenotype, seen previously for intracellular *Wolbachia* in C6/36 cells, indicating that the bacteria were unable to divide. In conclusion, we have developed a cell-free culture system in which *Wolbachia* replicate for up to 12 days, providing an *in vitro* tool to elucidate the biology of these endobacteria, e.g., cell division by using compounds that may not enter the C6/36 cells. A better understanding of *Wolbachia* biology, and in particular host-symbiont interactions, is key to the use of *Wolbachia* in vector control programs and to future drug development against filarial diseases.

## 1 Introduction

*Wolbachia* are intracellular Gram-negative alpha-proteobacteria found in arthropods and in some nematodes, including filarial nematode species pathogenic to humans (Taylor and Hoerauf, 2001; Fenn et al., 2006; Zug and Hammerstein, 2012). They reside in host-derived vesicles within cells of somatic tissues as well as the host germline through which they are transmitted vertically from the mother to the offspring (Casiraghi et al., 2007; Serbus and Sullivan, 2007). A common feature of endosymbiotic bacteria is the reduction of genome size due to the evolutionary adaptation to their host (Stepkowski and Legocki, 2001). This is also the case for *Wolbachia*, which possess a limited metabolic capacity. They lack almost all biosynthetic pathways to produce amino acids *de novo* and have retained almost only incomplete pathways for the synthesis of vitamins and cofactors, all of which are most probably provided by their host (Wu et al., 2004; Foster et al., 2005; Slatko et al., 2010).

*Wolbachia* endosymbionts of arthropods are largely facultative and often exhibit a parasitic association with their hosts (Werren et al., 2008). The stability of *Wolbachia* transmission is ensured by reproductive manipulations such as male-killing, feminization, parthenogenesis, and cytoplasmic incompatibility between infected and uninfected organisms (Fenn and Blaxter, 2006). Of note, benefits of an infection with *Wolbachia*, e.g., in terms of protection against different pathogens, have been reported (Hedges et al., 2008; Teixeira et al., 2008; Kambris et al., 2009; Moreira et al., 2009). Here, especially anti-viral effects have gained great interest as *Wolbachia* could be used to control vector-borne human diseases such as dengue fever (Blagrove et al., 2012; Velez et al., 2023).

*Wolbachia* of filarial nematodes are, in contrast to *Wolbachia* of arthropods, intrinsically tied to their host. Here, they are mutualistic endosymbionts that depend on compounds produced by the host, but in turn are believed to provide metabolites that cannot be synthesized by the nematodes *de novo*, e.g., heme, purines, pyrimidines, FAD, and riboflavin, essential for worm survival (Wu et al., 2004; Foster et al., 2005; Slatko et al., 2010). It was demonstrated that *Wolbachia* depletion by the antibiotic doxycycline leads to block in development, sterility, and death of adult filarial worms (Hoerauf et al., 2001; Taylor et al., 2012). Thus, filarial *Wolbachia* are an effective target for anti-filarial therapy.

The cultivation of *Wolbachia* as obligate intracellular bacteria is challenging. To date, filarial *Wolbachia* cannot be cultured *in vitro* (Slatko et al., 2014) and only a few culture systems exist, in which insect cell lines are stably infected with *Wolbachia* strains from arthropods (Fenollar et al., 2003a; McMeniman et al., 2008; Conceição et al., 2021). In these culture systems, *Wolbachia* are protected from the environment by at least three lipid membrane barriers: the insect cell membrane, vesicle membrane, and the *Wolbachia* cell membranes. Therefore, molecular biology techniques, e.g., genetic transformation, cannot be applied. Additionally, many molecules cannot pass the insect cell membrane, which hampers the elucidation of *Wolbachia* biology and its symbiosis with the host cell.

However, since *Wolbachia* are transmitted from somatic tissue to the germline (Frydman et al., 2006; Landmann et al., 2012), and also horizontally between host species (Dyson et al., 2002; White et al., 2017), even with plants as temporary hosts (Li et al., 2017), they require an extracellular stage (Nevalainen et al., 2023). This stage has been observed in the hemolymph of insects, foregut of ants, and pseudocoelomic cavity of filarial nematodes (Fischer et al., 2011; Andersen et al., 2012; Frost et al., 2014). Rasgon et al. (2006) showed that *Wolbachia* purified from insect cells could be maintained in cell-free culture medium for at least 1 week without loss of viability or infectivity. More recently, the metabolic activity of extracellular *Wolbachia* was measured via phenotypic microarrays over 4 days (Krafsur et al., 2020). However, *Wolbachia* in these cultures did not replicate outside the insect cell (Rasgon et al., 2006; Krafsur et al., 2020).

For a few intracellular bacteria, e.g., *Coxiella burnetii*, *Chlamydia trachomatis*, *Ehrlichia chaffeensis,* and *Anaplasma phagocytophilum*, cell-free culture systems were developed that support metabolic activity (Omsland et al., 2008, 2012; Eedunuri et al., 2018; Zhang et al., 2021). After further modifications, cell-free growth of *Coxiella burnetii* was made possible, accelerating genetic transformation (Omsland et al., 2009, 2011). An adapted medium allows for the non-antibiotic-based selection of genetic transformants (Sandoz et al., 2016).

In this study, we provide first evidence of *Wolbachia* replication in a host cell-free *in vitro* culture. Growth of *Wolbachia w*AlbB was observed when the medium was supplemented with total lysate from *Aedes albopictus* C6/36 insect cells. Furthermore, we could show that the necessary components for the replication of the endobacteria in cell-free medium are contained in the membrane fraction of the insect cell lysate and in fetal bovine serum (FBS).

## 2 Materials and methods

### 2.1 C6/36 insect cell culture

The *Aedes albopictus* C6/36 insect cell line, uninfected or infected with the *Wolbachia pipientis* supergroup B strain of *Aedes albopictus* (*w*AlbB), were cultured as previously described (Turner et al., 2006; Henrichfreise et al., 2009). Infected and uninfected C6/36 cells were grown at 26°C in 75 cm^2^ culture flasks (Greiner, Frickenhausen, Germany) with 15 mL standard medium consisting of Leibovitz’s L15 medium (Thermo Fisher Scientific, Waltham, Massachusetts, United States) supplemented with 5% fetal bovine serum (FBS; PAA Laboratories, Cölbe, Germany or PAN-Biotech, Aidenbach, Germany), 1% MEM non-essential amino acids (PAA Laboratories or Thermo Fisher Scientific), 2% tryptose phosphate broth (Sigma-Aldrich, Steinheim, Germany) and 1% penicillin/streptomycin (PAA Laboratories or Thermo Fisher Scientific). The standard 5% FBS in the culture media was changed to 20% to increase the percentage of infected cells (Clare et al., 2015) for later experiments as indicated.

### 2.2 Isolation of *Wolbachia* from insect cells

*Wolbachia* were purified from infected C6/36 cells either as described by Rasgon et al. (2006) or by an abbreviated protocol. The C6/36 cells were grown to ~90% confluence. Cells were harvested with a cell lifter (Corning, New York, United States) in 10 mL standard medium and lysed by vortexing with 100 sterile 3 mm borosilicate glass beads (Sigma-Aldrich) for 5 min. Cell debris was removed by centrifugation at 2,500 g for 10 min at 4°C (Heraeus Multifuge 4 KR, Heraeus, Hanau, Germany) and the supernatant was filtered through a 5 μm syringe filter (Sartorius, Göttingen, Germany). Our abbreviated protocol ended here, so that the insect cell lysate remained in the suspension. For purification following the procedure of Rasgon et al. (2006), *Wolbachia* were pelleted from the filtered supernatant by centrifugation at 18,400 g for 5 min at 4°C (Eppendorf Centrifuge 5,424 R, Eppendorf, Hamburg, Germany) on a 250 mM sucrose cushion (Sigma-Aldrich) and suspended in 10 mL standard medium. In contrast to Rasgon et al. (2006), the subsequent filtration was not performed with a 2.7 μm filter, but with a 1.2 μm syringe filter (Sartorius). The genomic DNA (gDNA) was isolated and the number of *Wolbachia* was determined by quantitative real-time PCR (qPCR) of the single-copy *Wolbachia* 16S rRNA gene as previously described (Makepeace et al., 2006).

### 2.3 Cell-free *Wolbachia* culture

To investigate the effect of insect cell lysate (see below) on isolated *Wolbachia,* the bacteria were purified from C6/36 either using the procedure published by Rasgon et al. (2006) or by the abbreviated procedure in which the insect cell lysate was retained. Isolated *Wolbachia* were diluted 1:5 in standard medium and incubated in 25 cm^2^ plug-sealed cell culture flasks (Greiner) at 26°C for 15 days. The number of *Wolbachia* was determined by 16S rRNA gene qPCR every one to three days. In the following assays, 200 μL cell-free *Wolbachia* extracted by the abbreviated protocol were incubated in F-bottom 96-well plates (Greiner) at 26°C for 12 days, and *Wolbachia* numbers were quantified by qPCR on day 0 and subsequently every three days.

For insect cell lysate titration assays, isolated *Wolbachia* (0.5–1.5 × 10^3^ 16S rRNA gene copies/μL) were added to total insect cell lysate equivalent to final concentrations of 0.95 × 10^6^ cells/mL, 1.9 × 10^6^ cells/mL, or 3.8 × 10^6^ cells/mL uninfected C6/36 cells as counted prior to lysis. Dilutions were prepared in standard medium. For *Wolbachia* cell number titration assays, different amounts of *Wolbachia* ranging from 10^2^ to 10^5^ 16S rRNA gene copies/μL were diluted in total cell lysate prepared from 0.95 × 10^6^ uninfected C6/36 cells and standard medium.

### 2.4 Preparation of insect cell lysate

#### 2.4.1 Total insect cell lysate

Insect cell lysate was generated from uninfected C6/36 cells. Briefly, cells were harvested in 10 mL standard medium and the amount of uninfected C6/36 cells was calculated using a Neubauer counting chamber (Laboroptik, Bad Homburg, Germany). Then, C6/36 cells were lysed by vortexing with 100 sterile 3 mm borosilicate glass beads for 5 min. Cell debris was removed by centrifugation at 2,500 g for 10 min at 4°C and the supernatant was filtered through a 5 μm syringe filter.

#### 2.4.2 Fractionation of insect cell lysate

Total insect cell lysate was fractionated by centrifugation at 20,000 g for 30 min at 4°C (Eppendorf Centrifuge 5,424 R) or at 100,000 g for 1 h at 4°C (Sorvall Discovery M120 SE, Sorvall, Waltham, USA), respectively. The supernatants containing microsomes and plasma membranes (Fraction 1) or the soluble cytoplasmic content (Fraction 3), respectively, were retained. Since ultracentrifugation could not be performed under sterile conditions, the supernatant obtained after centrifugation at 100,000 g for 1 h was sterile filtered through a 0.2 μm syringe filter (Sartorius), and the pellet was discarded. The pellet obtained after centrifugation at 20,000 g for 30 min containing nuclear debris and large organelles (Fraction 2) was dissolved in the same volume of standard medium as the starting volume of total lysate. Fractions were used for the preparation of cell-free *Wolbachia* cultures with a concentration of 0.5–1 × 10^3^ 16S rRNA gene copies/μL. The final concentration of fraction added to the culture was equivalent to 0.95 × 10^6^ C6/36 cells/mL as counted prior to lysis. For testing combinations of fractions, the final concentration of each fraction was 0.95 × 10^6^ cells/mL, and standard medium with 20% FBS was used. *Wolbachia* cultures with fractions were incubated at 26°C for 12 days. When supplementation with freshly prepared Fraction 1 on day 9 was tested, standard medium with 20% FBS was used, and growth was monitored until day 15.

#### 2.4.3 Insect cell lysate with and without FBS

*Wolbachia* were purified as described above. Two different insect cell lysates were prepared in cell culture medium with and without FBS. Prior to the preparation of insect cell lysate without FBS, the C6/36 cells were washed once in cell culture medium lacking FBS. Both lysates were centrifuged at 20,000 g for 30 min at 4°C and the supernatants were retained (Fraction 1). *Wolbachia* cultures containing Fraction 1 with and without FBS were incubated at 26°C for 12 days. A control containing *Wolbachia* incubated only in standard medium with FBS was included. The initial *Wolbachia* concentration was 0.1–1 × 10^4^ 16S rRNA gene copies/μL and the final concentration of Fraction 1 was equivalent to 0.95 × 10^6^ cells/mL.

### 2.5 Supplementation of cell-free culture with cholesterol

Cell-free *Wolbachia* cultures were prepared as described above with 0.5–1 × 10^3^ 16S rRNA gene copies/μL isolated *Wolbachia* and Fraction 1 from insect cell lysate equivalent to 0.95 × 10^6^ cells/mL diluted in standard medium with 20% FBS, cultured in 96-well plates at 26°C and supplemented with 0.1 or 1 mg/mL water-soluble cholesterol (Sigma-Aldrich) for 12 days.

### 2.6 Infection of C6/36 insect cells with *Wolbachia* from cell-free culture

Cell-free *Wolbachia* cultures were prepared as described above with 0.5 × 10^3^ 16S rRNA gene copies/μL isolated *Wolbachia* and Fraction 1 from insect cell lysate equivalent to 0.95 × 10^6^ cells/mL diluted in standard medium and cultured in 96-well plates at 26°C for 12 days. After 9 days, uninfected C6/36 cells were seeded in an F-bottom 24-well plate (Greiner) with 10^5^ cells/well in triplicate. On day 12, the medium was removed from the uninfected C6/36 cells and 750 μL of the cell-free *Wolbachia* culture were added, corresponding to a multiplicity of infection (MOI) of 14. As a negative control, *Wolbachia* were heated at 95°C for 10 min, before adding them to the uninfected C6/36 cells. The cells, covered with cell-free *Wolbachia* culture, were centrifuged at 2,000 g for 1 h at 15°C and subsequently incubated at 26°C overnight. On the next day, cells were transferred into an F-bottom 6-well plate (Greiner) containing 1.5 mL standard medium with 10% FBS and incubated at 26°C. After 6 days, the C6/36 cells were harvested in fresh standard medium and transferred into an 8-well culture slide (BD Falcon, Corning, United States). Additionally, samples were taken for qPCR. C6/36 cells were grown on culture slides for 1 day. *Wolbachia* infection was subsequently examined by immunofluorescence microscopy using rabbit anti-*w*PAL primary antiserum (1:1,000 in PBST; Taylor Laboratory, Liverpool School of Tropical Medicine, Liverpool, UK) and a goat anti-rabbit Alexa 488-conjugated secondary antibody (1:200 in PBST; Thermo Fisher Scientific) and counterstained with 0.25 μg/mL DAPI (Sigma-Aldrich) as described previously (Turner et al., 2009; Vollmer et al., 2013). Cells were then analyzed with a Zeiss Axio Observer.Z1 fluorescence microscope (Carl Zeiss AG, Oberkochen, Germany) at the respective wavelengths.

### 2.7 Quantitative real-time PCR

gDNA was extracted from 200 μL using the QIAamp DNA Mini Kit (Qiagen, Hilden, Germany) following the manufacturer’s instructions for DNA purification from blood or body fluids with an adjusted elution volume of 50 μL in a QIAcube robotic workstation (Qiagen). *Wolbachia* cell numbers were calculated by quantification of 16S rRNA gene copies by qPCR as previously described (Makepeace et al., 2006) using the HotStar Taq Polymerase Kit (Qiagen). A qPCR reaction contained 1x HotStar Taq polymerase buffer, 3 mM MgCl_2_, 200 μM dNTPs, 0.2 μL SYBR Green (1,000-fold diluted in DMSO; Fermentas, St. Leon-Rot, Germany), 0.5 μM 16S rRNA primers (forward: 5’-TTGCTATTAGATGAGCCTATATTAG-3′, reverse: 5’-GTGTGGCTGATCATC CTCT-3′; Microsynth, Balgach, Switzerland), 0.5 U HotStar Taq polymerase and 2 μL of extracted gDNA (1:20 diluted in AE buffer for cell culture samples, undiluted for cell-free samples). qPCR conditions included a heat activation step at 95°C for 15 min followed by 45 cycles of 95°C for 10 s, 55°C for 15 s, and 72°C for 20 s. Actin qPCRs were applied to control for C6/36 replication (Henrichfreise et al., 2009). For actin qPCRs, a reaction mixture contained 1x HotStar Taq polymerase buffer, 1 mM MgCl_2_, 200 μM dNTPs, 0.2 μL SYBR Green (1,000-fold diluted in DMSO), 0.3 μM actin primers (forward: 5’-ACGAACTGGGACGATATGGA-3′, reverse: 5’-GCCTCTGTCAGGAGAACTGG-3′; Microsynth, Balgach, Switzerland), 0.5 U HotStar Taq polymerase and 2 μL of extracted gDNA (1:20 diluted in AE buffer for cell culture samples, undiluted for cell-free samples). qPCR conditions included a heat activation step at 95°C for 15 min followed by 45 cycles of 95°C for 10 s, 57°C for 15 s, and 72°C for 20 s. Melt curve analysis showed a specific peak for all positive samples. Data were analyzed using Rotor-Gene 6,000 software version 1.7 (Corbett Life Sciences, Sydney, Australia). The fold change in 16S rRNA gene and actin copies is calculated by dividing the value of each time point by the mean copy number at D0 and indicates replication of *Wolbachia* and C6/36 cells, respectively.

### 2.8 Fluorescence microscopy of antibiotic-treated cell-free *Wolbachia*

Cell-free *Wolbachia* cultures were prepared as described above with 0.5 × 10^3^ 16S rRNA gene copies/μL isolated *Wolbachia* and Fraction 1 from insect cell lysate equivalent to 0.95 × 10^6^ cells/mL diluted in standard medium with 20% FBS, cultured in 96-well plates at 26°C for 12 days and treated with 512 μg/mL fosfomycin (Infectofos, InfectoPharm, Heppenheim, Germany) daily or every three days with ampicillin (Sigma-Aldrich), bacitracin (AppliChem, Darmstadt, Germany), or vancomycin (Sigma-Aldrich). 50 μL of cell-free *Wolbachia* were dried on a microscopy slide and stained as described for the infection experiment. Cell diameter of fosfomycin-treated cells was measured based on the *w*Pal staining using ImageJ (Version 2.0.0-rc-43/1.50e, https://imagej.nih.gov/ij/).

### 2.9 Statistical analysis

For statistical analysis, GraphPad Prism version 10.1.2 for Windows (GraphPad Software, Boston, Massachusetts United States, www.graphpad.com) was used.

## 3 Results

### 3.1 *Wolbachia* replicate under cell-free conditions

**Figure 1:**
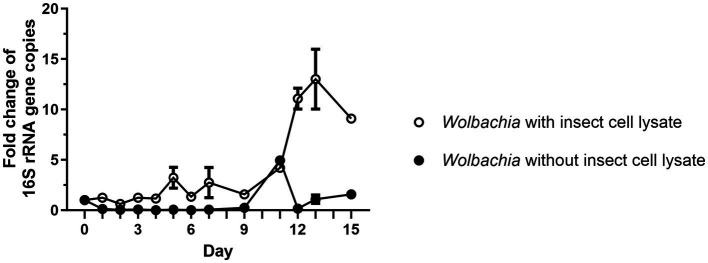
Isolated *Wolbachia* replicate in medium when the C6/36 cell membranes are retained. *Wolbachia* were purified from C6/36 cells via ultracentrifugation (Rasgon et al., 2006), or were purified by an abbreviated protocol that retained more of the insect cell lysate. Cell-free cultures were incubated at 26°C for 15 days and samples were taken every one to three days. *Wolbachia* were quantified by qPCR of the 16S rRNA gene. Copy numbers were normalized to day 0. Data were pooled from two independent experiments. For days 2, 4, 6 (experiment 1) and days 9, 11, 15 (experiment 2), the data from only one experiment is shown. For the other days, the mean ± SEM of 2–5 wells is shown.

As a first step toward establishing an insect cell-free culture of replicating *Wolbachia*, it was investigated whether lysate from disrupted host cells is sufficient for wolbachial growth. For this, two different cell-free *Wolbachia* suspensions were prepared. The first suspension contained *Wolbachia* purified according to the procedure published by Rasgon et al. (2006). The second suspension contained *Wolbachia* purified according to an abbreviated protocol in which the high-speed centrifugation on a sucrose cushion and the subsequent filtration step through a 1.2 μm filter were omitted, which retained more of the insect cell lysate. A 1:5 dilution of each suspension in standard medium was incubated in 25 cm^2^ cell culture flasks at 26°C for up to 15 days. Samples were removed every one to three days (exact timing is shown in figures) and the number of *Wolbachia* was determined by qPCR. Gene copy numbers were normalized to the counts on day 0 of the culture. In the cell-free *Wolbachia* culture with retained insect cell lysate, 16S rRNA gene copies increased 3.2-fold by day 5 and 13-fold by day 13 (Figure 1). In contrast, the number of cell-free *Wolbachia*, purified as described by Rasgon et al. (2006) and thus without insect cell lysate, decreased by 88% from day 0 to day 3 and remained unchanged until day 9. An apparent increase was observed on day 11; however, considering the absence of subsequent replication and this time point being a single replicate, this data point was considered an outlier. *Actin* copy numbers were monitored to exclude the possibility that intact C6/36 cells remained in the culture; no increase in *actin* copy number was measured (Supplementary Figure S1). In the following, *Wolbachia* were purified using the abbreviated protocol.

We wanted to further characterize the conditions for cell-free growth of *Wolbachia* to enable consistent assays. In addition, faster growth of cell-free *Wolbachia* would be desirable to allow easy application, e.g., for antibiotic assays. Since the starting amount of C6/36 cells was not measured, our next step was to first determine the optimal amount of insect cell lysate.

### 3.2 *Wolbachia* replication is inversely dependent on the amount of lysate from uninfected C6/36 cells

**Figure 2:**
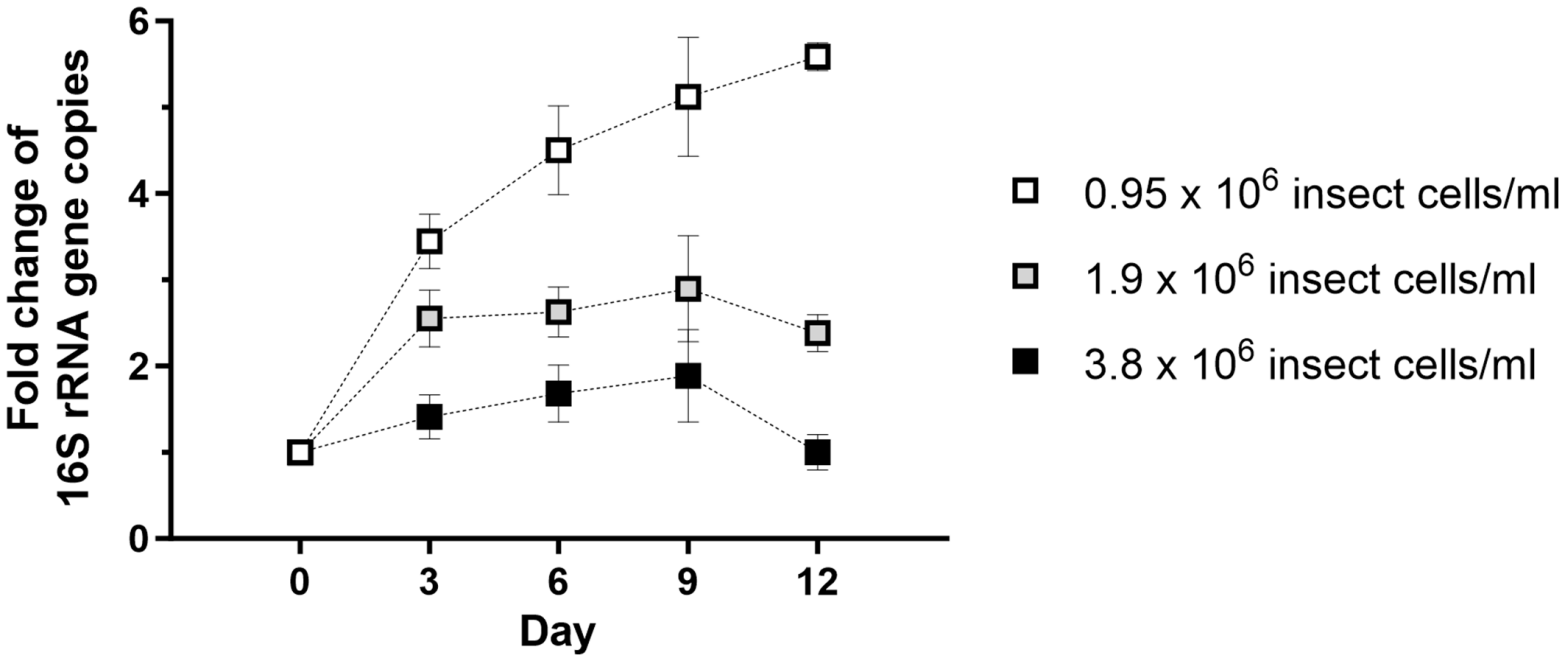
*Wolbachia* replication in cell-free culture is dose-dependent on the amount of C6/36 cell lysate. Total cell lysate from uninfected C6/36 cells was prepared from the depicted cell numbers determined in a Neubauer counting chamber prior to cell lysis. Purified *Wolbachia* (0.5–1.5 × 10^3^ 16S rRNA gene copies/μL) were incubated at 26°C for 12 days with the three indicated dilutions of insect cell lysate. Growth was monitored by 16S rRNA gene qPCR every three days and data were normalized to day 0. Data were pooled from two independent experiments. For every time point, the mean ± SEM of six wells is shown.

Purified *Wolbachia* (0.5–1.5 × 10^3^ 16S rRNA gene copies/μL) were incubated with different dilutions of total cell lysate prepared from uninfected C6/36 cells. *Wolbachia* replication was detected in all dilutions of cell lysate, with the highest overall copy number of the 16S rRNA gene on day 9 (Figure 2). In lysate equivalent to 3.8 × 10^6^ cells/mL, *Wolbachia* numbers increased up to 1.9-fold compared to day 0. In more diluted insect cell lysates, the *Wolbachia* replication rate was even higher, achieving an up to 2.9-fold increase with lysate from 1.9 × 10^6^ cells/mL and up to 5.1-fold with lysate from 0.95 × 10^6^ cells/mL. *Wolbachia* growth was achieved from day 0 to day 9 when using the two higher-concentrated lysates, whereas lower *Wolbachia* concentrations were measured on day 12. For the lowest lysate concentration, growth was also observed to day 12. Based on these results, cell lysate prepared from uninfected C6/36 cells equivalent to 0.95 × 10^6^ cells/mL was used for further experiments.

### 3.3 Replication in cell-free medium is *Wolbachia* density-dependent

**Figure 3:**
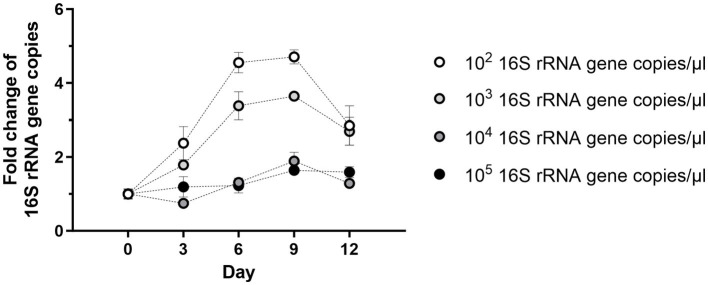
Starting density of *Wolbachia* influences cell-free replication. Different starting concentrations of *Wolbachia* were incubated with total insect cell lysate (equivalent to 0.95 × 10^6^ uninfected C6/36 cells) at 26°C for 12 days. Growth was monitored by 16S rRNA gene qPCR every three days and data were normalized to day 0. The graph is representative of two independent experiments. For every time point, the mean ± SEM of three wells is shown.

Next, the optimal initial density of *Wolbachia* for growth in cell-free culture was titrated. *Wolbachia* were purified from infected C6/36 cells and total cell lysate from uninfected C6/36 cells was prepared. Decreasing concentrations of *Wolbachia* from 10^5^ to 10^2^ 16S rRNA gene copies/μL were suspended in standard medium containing total insect cell lysate. In cell-free culture containing high *Wolbachia* counts of 10^5^ or 10^4^ 16S rRNA gene copies/μL, the counts slightly increased 1.6- and 1.9-fold, respectively, until day 9 (Figure 3). In contrast, cultures containing 10^3^ or 10^2^ 16S rRNA gene copies/μL had higher replication rates between days 0 and 9, increasing 3.6- and 4.7-fold, respectively. At all concentrations, *Wolbachia* numbers decreased to day 12. Therefore, unless otherwise stated, initial *Wolbachia* concentrations between 10^2^ and 10^3^ 16S rRNA gene copies/μL were used for further experiments.

### 3.4 Insect cell membranes are essential for *Wolbachia* replication

*Wolbachia* replication might be dependent on soluble signaling molecules or growth factors provided by the C6/36 cells. As a first step to verify this possibility, insect cell lysate was separated by centrifugation to achieve a rough fractionation of C6/36 cell components (Lodish et al., 2000). For this, insect cell lysate was centrifuged at 20,000 g for 30 min or ultracentrifuged at 100,000 g for 60 min. The supernatant after 20,000 g centrifugation containing cytosol, microsomes, and plasma membranes of the C6/36 cells was retained (Fraction 1), and the corresponding pellet containing nuclear debris and large cell organelles was resuspended in standard medium (Fraction 2). The supernatant after ultracentrifugation containing soluble cytoplasmic contents was also retained (Fraction 3). All three fractions equivalent to 0.95 × 10^6^ cells/mL were incubated separately with 10^3^ 16S rRNA gene copies/μL of purified *Wolbachia*. As controls, *Wolbachia* were grown in total insect cell lysate and in standard medium without lysate.

**Figure 4:**
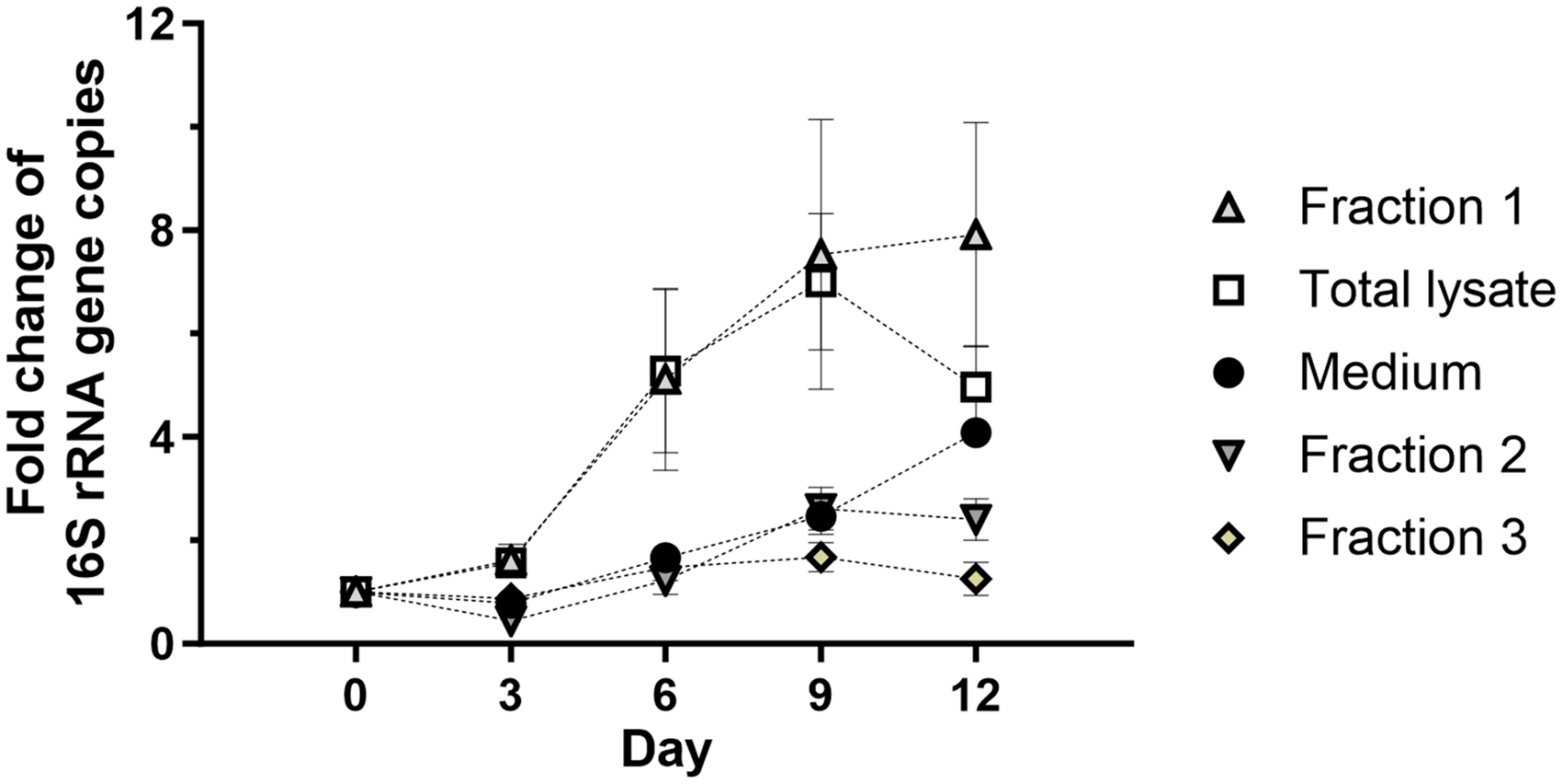
The cell membrane-containing fraction from C6/36 cells is required for *Wolbachia* replication in a cell-free culture. Total cell lysate was prepared from 0.95 × 10^6^ uninfected C6/36 cells. A portion of cell lysate was fractionated by centrifugation at 20,000 g for 30 min or 100,000 g for 60 min. *Wolbachia* were incubated in the supernatant retained after 20,000 g centrifugation (Fraction 1, microsomes and membranes), the corresponding pellet resuspended in cell culture medium (Fraction 2, nuclear debris and organelles), or the supernatant retained after 100,000 g (Fraction 3, soluble cytoplasmic molecules) at 26°C for 12 days. Growth was compared to reactions containing total insect cell lysate or medium alone. The initial concentration of *Wolbachia* was 10^3^ 16S rRNA gene copies/μL. Growth was monitored by 16S rRNA gene qPCR every three days and data were normalized to day 0. Data were pooled from two independent experiments. For every time point, the mean ± SEM of six wells is shown, except for the medium group for which the mean ± SEM of three wells is shown.

*Wolbachia* incubated with Fraction 1 had equivalent replication as *Wolbachia* incubated with total insect cell lysate, reaching 7-fold mean replication on day 9 compared to day 0. However, the group with Fraction 1 showed growth until day 12 (Figure 4). *Wolbachia* incubated in medium alone or supplemented with Fraction 2 or Fraction 3 had similar growth curves until day 9 when they had replicated 2- to 3-fold. The medium group continued to replicate until day 12, never reaching more than 50% growth compared to the culture with Fraction 1, while the other two had a slight decrease. To investigate whether Fraction 2 or 3 contain compounds with an inhibitory effect on extracellular wolbachial growth, combinations of all fractions were tested. Replication rates were lower for Fraction 1 in combination with Fraction 2 or Fraction 3 than for Fraction 1 alone, indicating inhibitory effects (Supplementary Figure S2). Therefore, for all further experiments, cell-free *Wolbachia* cultures were supplemented with Fraction 1 (= membrane fraction).

**Figure 5:**
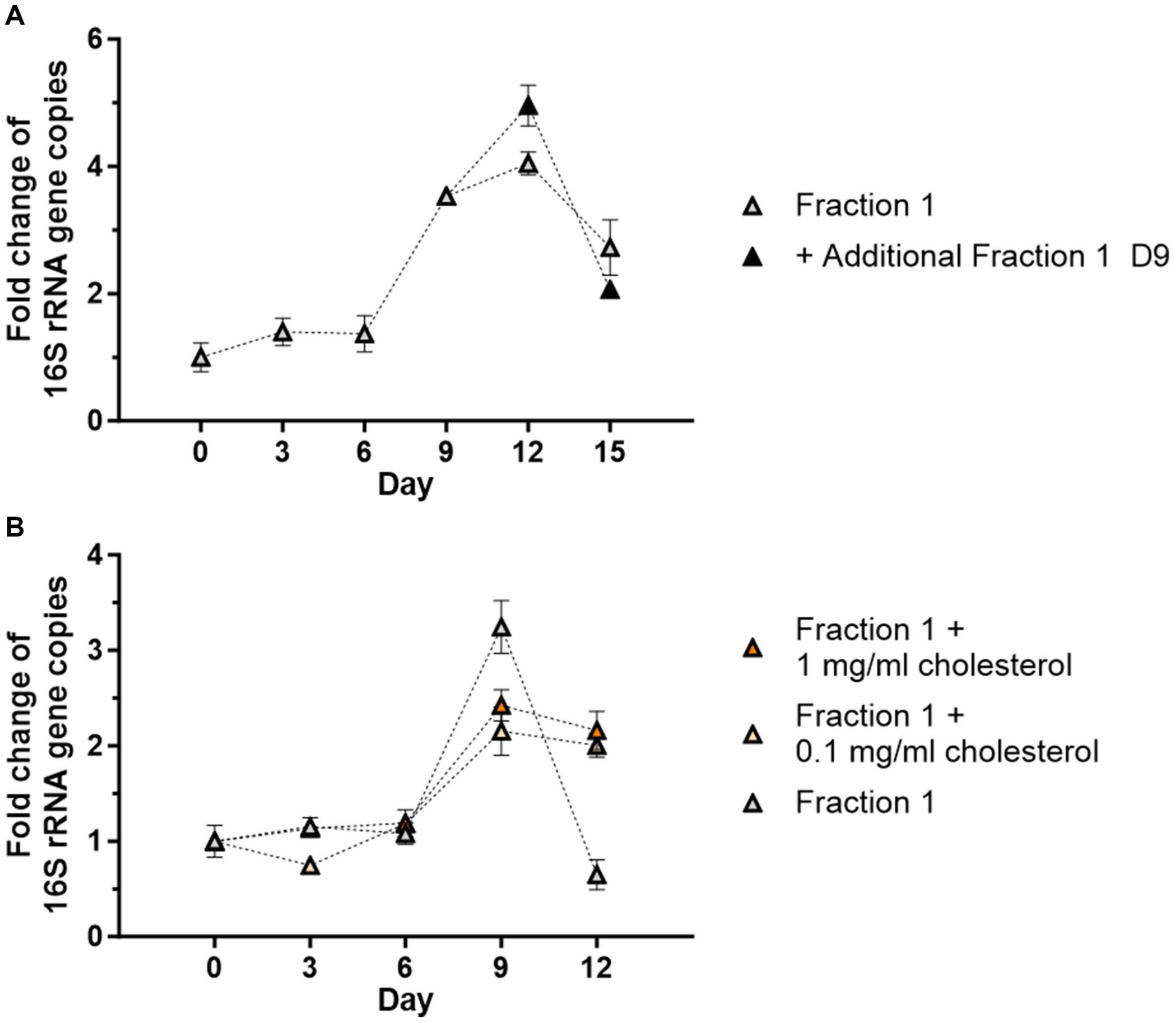
Addition of fresh Fraction 1 or cholesterol do not support cell-free replication. **(A)** Cell-free *Wolbachia* (2 × 10^2^ 16S rRNA gene copies/μL) were incubated with Fraction 1 from uninfected C6/36 cells (equivalent to 0.95 × 10^6^ cells/mL) at 26°C for 15 days. On day 9, fresh Fraction 1 was added to half of the remaining wells. Growth was monitored by 16S rRNA gene qPCR every three days and data were normalized to day 0. The graph is representative of two independent experiments. For every time point, the mean ± SEM of three wells is shown. **(B)** Cell-free *Wolbachia* (0.5 × 10^3^ 16S rRNA gene copies/μL) were incubated with Fraction 1 from uninfected C6/36 cells (equivalent to 0.95 × 10^6^ cells/mL) with or without water-soluble cholesterol (0.1 or 1 mg/mL) at 26°C for 12 days. Growth was monitored by 16S rRNA gene qPCR every three days and data were normalized to day 0. For every time point, the mean ± SEM of six wells is shown.

To further extend cell-free growth, freshly prepared Fraction 1 was applied to the culture on day 9 and replication was monitored via qPCR until day 15. Growth rates were slightly higher in the supplemented group on day 12 (5-fold) than in the standard cell-free culture (4-fold), but growth was not prolonged since both groups showed a decrease to day 15 (Figure 5A). A second supplementation with fresh Fraction 1 on day 12 also did not extend cell-free *Wolbachia* replication (data not shown). Insufficient amounts of cholesterol were also considered to be a potential limiting factor of cell-free *Wolbachia* growth. Thus, freshly prepared water-soluble cholesterol was added to the cell-free culture with Fraction 1, but no increase in replication was observed (Figure 5B).

### 3.5 FBS is essential for *Wolbachia* replication

**Figure 6:**
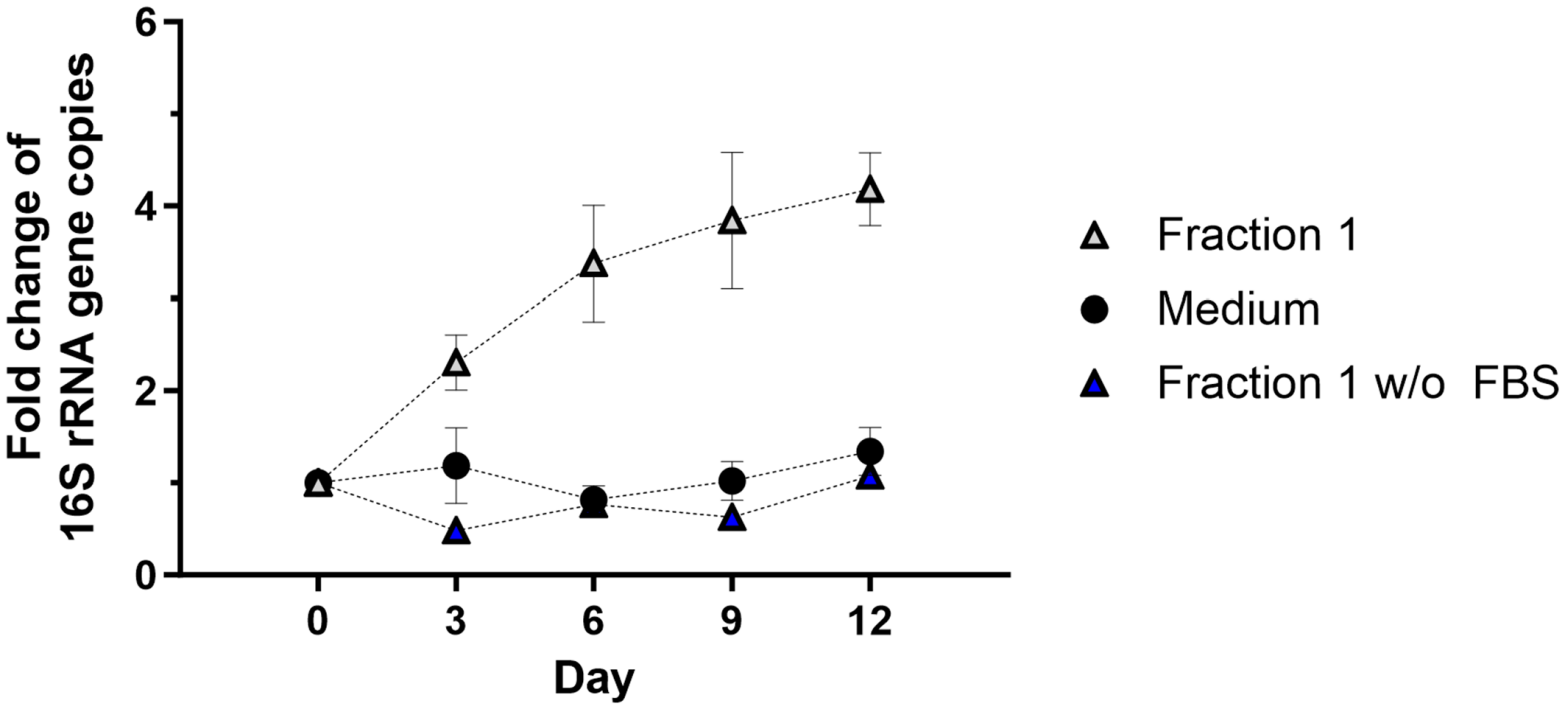
FBS is required for *Wolbachia* replication in a cell-free culture. Cell-free *Wolbachia* (0.1–1 × 10^4^ 16S rRNA gene copies/μL) were incubated with Fraction 1 from uninfected C6/36 cells (equivalent to 0.95 × 10^6^ cells/mL) harvested in cell culture medium either with or without FBS and incubated at 26°C for 12 days. Growth was monitored by 16S rRNA gene qPCR every three days and data were normalized to day 0. Data were pooled from two independent experiments. For every time point, the mean ± SEM of six wells is shown.

The growth rate of C6/36 insect cells in cell culture medium is slower in FBS-free medium (Kuno, 1983). The exact components of FBS are not known but many hormones, growth factors, and nutrients are provided with the serum. Thus, it was investigated whether FBS also supports or is necessary for *Wolbachia* replication in the cell-free system. *Wolbachia* grown in standard medium supplemented with Fraction 1 replicated as seen before, reaching a 4.2-fold increase on day 12 (Figure 6). In contrast, *Wolbachia* incubated in medium barely replicated, with a 1.3-fold increase on day 12. No increase of cell-free *Wolbachia* was detected when grown in FBS-free medium supplemented with Fraction 1 derived from uninfected C6/36 cells also harvested in FBS-free medium. These results show that both FBS and Fraction 1 are necessary for replication of cell-free *Wolbachia*; one without the other is not sufficient.

### 3.6 *Wolbachia* from cell-free culture can infect C6/36 cells

**Figure 7:**
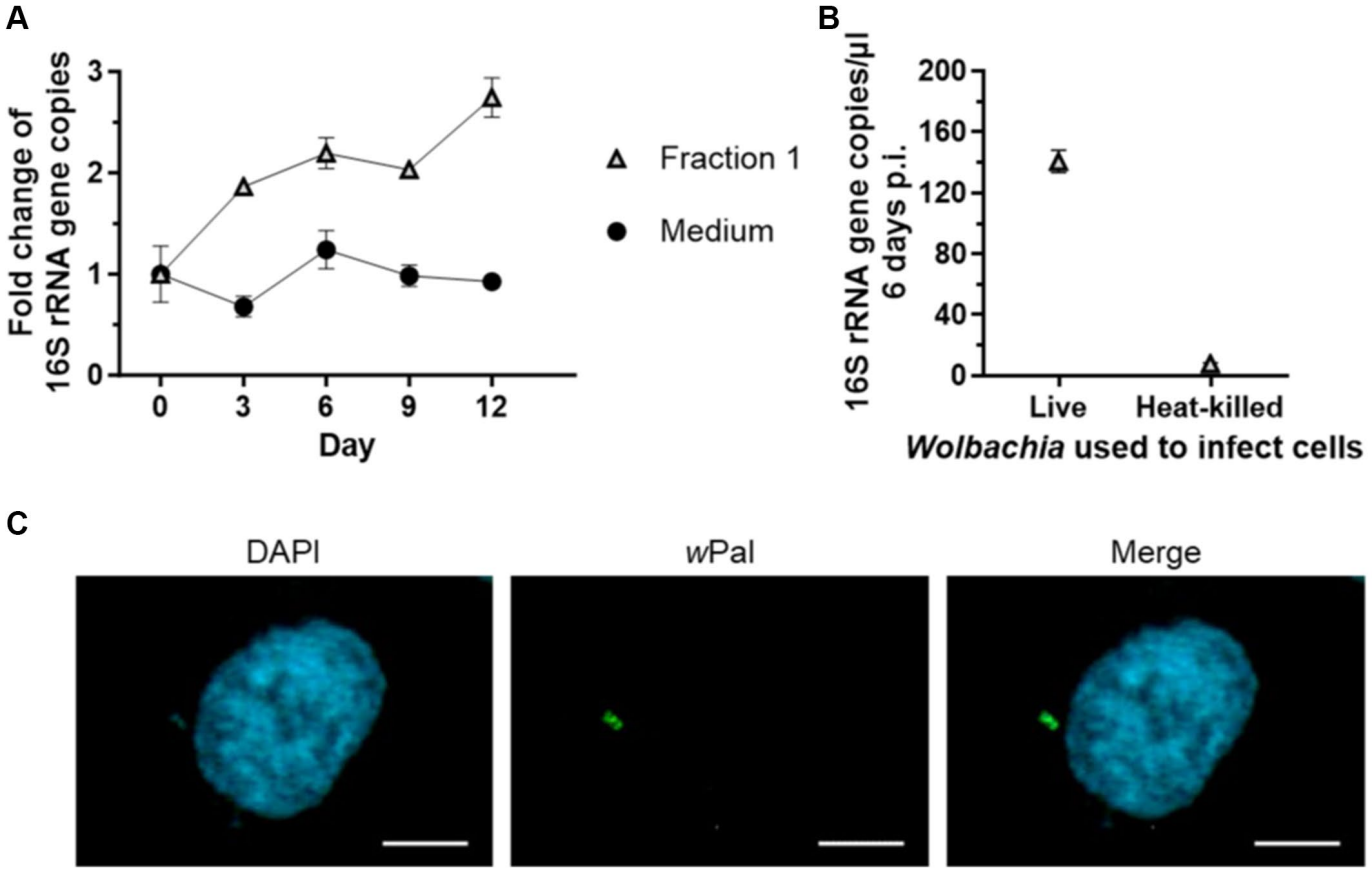
Cell-free cultured *Wolbachia* infect uninfected C6/36 cells. **(A)** Cell-free *Wolbachia* (0.5 × 10^3^ 16S rRNA gene copies/μL) were incubated with Fraction 1 from uninfected C6/36 cells (equivalent to 0.95 × 10^6^ cells/mL) at 26°C for 12 days. Growth was monitored by 16S rRNA gene qPCR every three days. For every time point, the mean ± SEM of three wells is shown. **(B)** On day 12, 750 μL of this cell-free *Wolbachia* culture were added to uninfected C6/36 cells grown in a 24-well plate. As a negative control, *Wolbachia* were heat-killed at 95°C for 10 min prior to addition to the uninfected C6/36 cells. After centrifugation at 2,000 g for 1 h at 15°C, the plate was incubated overnight at 26°C. On the next day, the medium was removed and fresh cell culture medium was added. On day 6 post-infection, three samples were taken for 16S rRNA gene qPCR of C6/36 cells infected with *Wolbachia* and with heat-killed *Wolbachia* (mean ± SEM). Data are representative of two experiments. **(C)** Six days post-infection, C6/36 cells were grown on culture slides for 1 day and subsequently examined with a Zeiss Axio Observer.Z1 fluorescence microscope using immunofluorescence microscopy with *w*PAL anti-serum and an Alexa 488-conjugated secondary antibody (green, *Wolbachia*) and counterstained with DAPI (blue). Scale bar: 5 μm.

Rasgon et al. (2006) and Nevalainen et al. (2023) demonstrated that purified *Wolbachia* can infect uninfected insect cells. Therefore, we wanted to determine if *Wolbachia* that replicated in our cell-free culture system had maintained the infective phenotype. The infectivity of *Wolbachia* was examined by infecting uninfected C6/36 cells with *Wolbachia* grown in cell-free culture for 12 days (Figure 7A). For cell-free cultured *Wolbachia* incubated with Fraction 1, the number of *Wolbachia* increased 2.7-fold to day 12 (Figure 7A). *Wolbachia* from day 12 of this cell-free culture were used to infect C6/36 cells. Six days post-infection, ~140 16S rRNA gene copies/μL were measured in the cell culture (Figure 7B). In contrast, only eight 16S rRNA gene copies/μL were detected in C6/36 cells infected with heat-killed *Wolbachia* from the same cell-free culture. Immunofluorescence microscopy using a *Wolbachia*-specific antiserum against *w*Pal confirmed the presence of *Wolbachia* in the C6/36 cell culture 7 days post-infection when infected with live *Wolbachia* (Figure 7C).

### 3.7 *Wolbachia* replication rate is lower in cell-free culture

**Figure 8:**
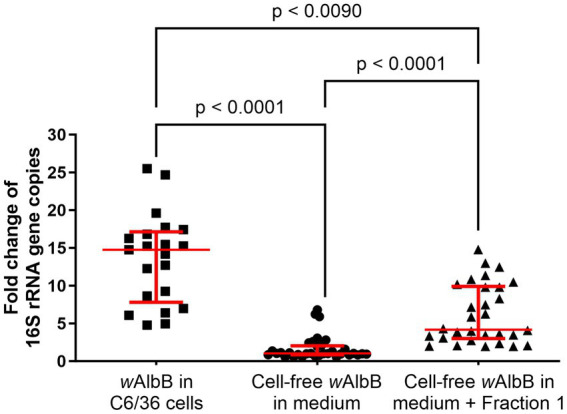
Variation in growth between *Wolbachia* cultured in cell-free medium ± Fraction 1 from C6/36 cell lysate compared to standard C6/36 cell culture. The replication of *Wolbachia* in cell-free culture with and without Fraction 1 from insect cell lysate or in C6/36 cells was compared on day 9, combining data from at least 20 independent experiments performed in duplicates (growth in C6/36 cells) or triplicates (cell-free growth), respectively. Cell-free *Wolbachia* with initial concentrations of 10^2^–10^3^ 16S rRNA gene copies/μL were incubated with Fraction 1 from uninfected C6/36 cells (equivalent to 0.95 × 10^6^ cells/mL). *Wolbachia* in C6/36 cells had initial concentrations of 10^3^–10^4^ 16S rRNA gene copies/μL. Each dot represents one experiment. The median with interquartile range is shown (red lines). Statistical differences were determined using a Kruskal-Wallis test followed by a post-hoc Dunn’s multiple comparisons test using GraphPad Prism 10.

By supplementing standard medium with Fraction 1, *Wolbachia* were able to replicate for at least 9 days. To determine the stability and growth efficiency of *Wolbachia* in the cell-free culture system, growth rates of *Wolbachia* cultured with and without Fraction 1 were compared to *Wolbachia* cultured within the C6/36 cell line. For each culture system, the fold increase of 16S rRNA gene copies on day 9 was compared. Growth rates significantly differed between the groups (Figure 8). *Wolbachia* residing in C6/36 cells had a median growth rate of 14.8 (range 4.8–25.5; mean: 13.6). Cell-free *Wolbachia* cultured in standard medium had a median growth rate of 1.0 (range 0.6–6.8; mean: 1.7). Cell-free *Wolbachia* cultured in standard medium supplemented with Fraction 1 had a median growth rate of 4.2 (range 2–14.8; mean: 6.4).

### 3.8 Cell-free cultured *Wolbachia* are sensitive to fosfomycin treatment

**Figure 9:**
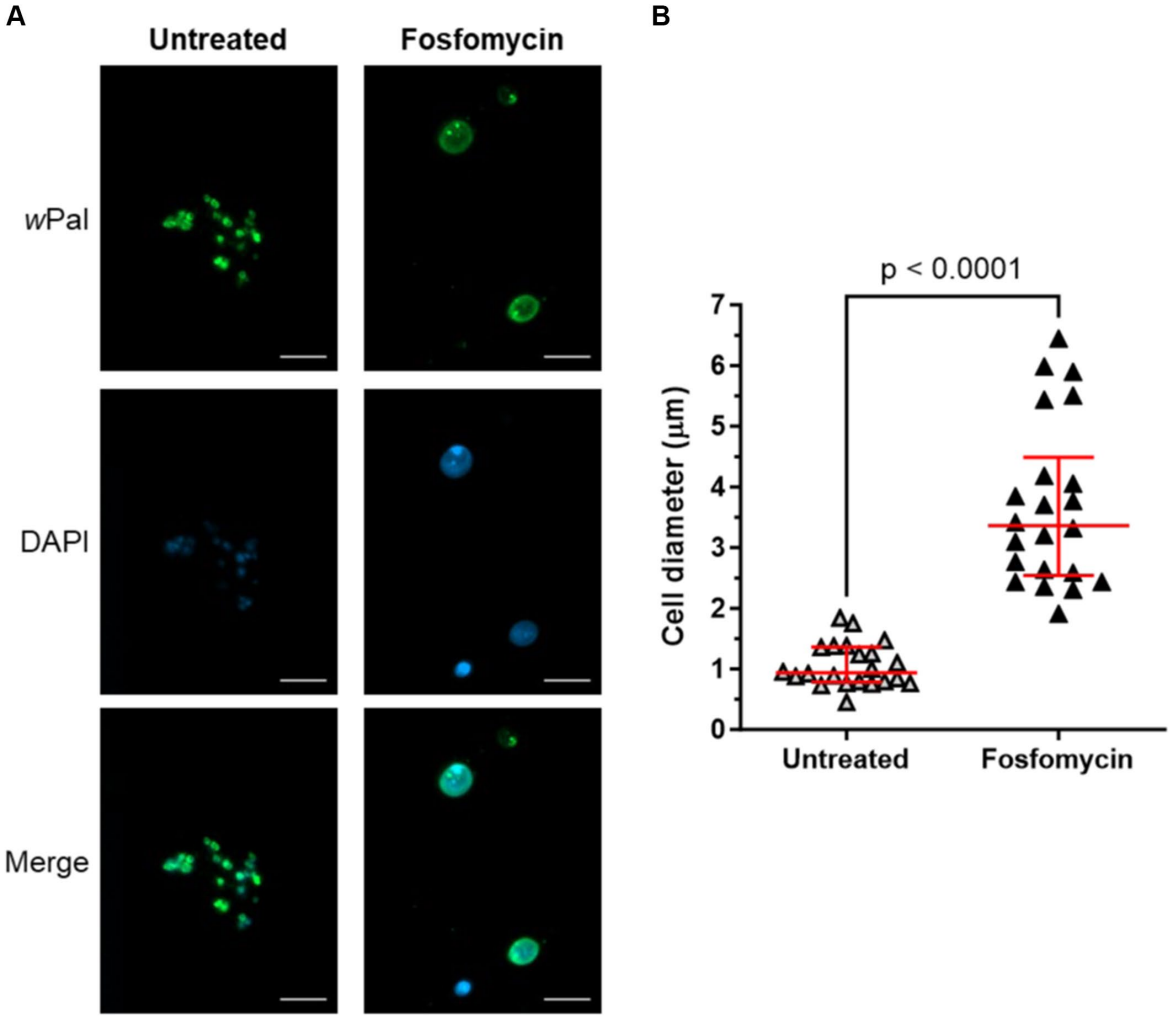
Cell-free cultured *Wolbachia* are sensitive to fosfomycin treatment. Cell-free *Wolbachia* (0.5 × 10^3^ 16S rRNA gene copies/μL) were incubated with Fraction 1 from uninfected C6/36 cells (equivalent to 0.95 × 10^6^ cells/mL) at 26°C for 12 days, with or without daily 512 μg/mL fosfomycin treatment. **(A)** Cells were fixed and visualized by immunofluorescence microscopy using *w*PAL anti-serum and an Alexa 488-conjugated secondary antibody (green, *Wolbachia*) and counterstained with DAPI (blue). Scale bar: 5 μm. **(B)** Cell diameter (median with IQR, red lines) was measured with ImageJ based on the *w*Pal staining from three independent assays (*n* = 22). Statistical differences were determined using a Mann–Whitney test using GraphPad Prism 10.

It has been shown that *Wolbachia* are sensitive to fosfomycin (Henrichfreise et al., 2009), a specific inhibitor of MurA that catalyzes the first dedicated step of lipid II biosynthesis. Treatment of *Wolbachia*-infected C6/36 cells with fosfomycin resulted in fewer and enlarged *Wolbachia* cells, demonstrating that the cell wall precursor lipid II is necessary for cell division in *Wolbachia* (Vollmer et al., 2013). To confirm that cell-free cultivated *Wolbachia* are suitable for antibiotic studies, e.g., to understand the reduced cell division machinery encoded in the genome, the phenotype of fosfomycin-treated endobacteria was analyzed via immunofluorescence microscopy using anti-*w*PAL. Untreated cell-free *Wolbachia* had a median (IQR) cell diameter of 0.94 μm (0.78–1.36 μm), whereas fosfomycin-treated *Wolbachia* were significantly larger with 3.36 μm (2.54–4.49 μm) (Figure 9). We only observed these enlarged cells when we also detected wolbachial replication via qPCR of the 16S rRNA gene. In contrast, the other cell wall biosynthesis-inhibiting antibiotics tested, i.e., ampicillin, bacitracin, and vancomycin did not affect the phenotype of cell-free *Wolbachia* (Supplementary Figure S3).

## 4 Discussion

*In vitro* culture systems of *Wolbachia* necessary for the elucidation of their biology are few and only *Wolbachia* strains naturally occurring in arthropods have been successfully cultured in insect cell lines (Fenollar et al., 2003a; McMeniman et al., 2008), while all attempts to culture *Wolbachia* of filarial nematodes have failed (McNulty et al., 2010; Slatko et al., 2014; Marriott et al., 2023). Molecular biological techniques are mostly impossible to apply to *Wolbachia* cultured in insect cell lines, e.g., genetic transformation or treatment of *Wolbachia* with large antibiotics such as aminoglycosides, polymyxins, lipo-and glycopeptide antibiotics that might not pass the insect cell membranes. Therefore, the extracellular cultivation of *Wolbachia* would provide an excellent tool for understanding the biology and symbiosis of *Wolbachia*. However, *Wolbachia* purified from insect cells have only been maintained without replication in cell-free cultures (Rasgon et al., 2006; Krafsur et al., 2020). Further attempts regarding *ex vivo* growth failed, but some components were advantageous regarding survival of *Wolbachia*, e.g., compatible solutes, actin, and mammalian blood (Uribe-Alvarez et al., 2018).

For other intracellular species such as *Coxiella burnetii* a complex medium has been designed in which cell-free growth occurred (Omsland et al., 2009). However, contrary to *Wolbachia*, *Coxiella burnetii* exhibit a less symbiotic interaction with their host cell and can even persist in an extracellular environment (Heinzen et al., 1999). Furthermore, attempts to generate a complex medium for cell-free growth of *Chlamydia*, which have a lifestyle that is more similar to that of *Wolbachia*, were unsuccessful (Omsland et al., 2012). This points out the complexity of cell-free growth of obligate intracellular bacteria that are tightly associated with their host. Compared to *Coxiella burnetii*, *Wolbachia* and *Chlamydia* possess a substantially reduced genome, which might make cell-free growth even more difficult.

In the present study, we could demonstrate that *Wolbachia* were not only viable when maintained in a cell-free culture, but underwent replication when insect cell lysate from uninfected C6/36 cells was added to the medium. In some experiments, a slight increase in *Wolbachia* numbers was observed in standard cell culture medium without insect cell lysate, but it never reached the levels observed when supplementing the cell-free medium, and in most cases no growth was detected. It is possible that the *Wolbachia* suspension generated from infected C6/36 cells contained sufficient components that allowed for a weak replication rate.

Viability and infectivity of *Wolbachia* from a 12-day-old cell-free *Wolbachia* culture were confirmed by infecting uninfected C6/36 cells, with *Wolbachia* DNA and intracellular *Wolbachia* detectable six and seven days post-infection. With a maximum of 1–2 *Wolbachia* per C6/36 cell, the insect cells were considerably less infected than the Aa23 and JW18 cells of Rasgon et al. (2006) and Nevalainen et al. (2023), respectively. While this could be supported by different uptake efficiencies of the *Wolbachia* subspecies and insect cells, it is most likely explained by the different MOIs used for infection. We used an MOI of 14, whereas Rasgon et al. (2006) used an MOI of 2,600 and Nevalainen et al. (2023) used an MOI of 20:1 host cell equivalents (i.e., the *Wolbachia* contents from 20 infected cells per seeded uninfected cell). Our MOI was low because of the low *Wolbachia* numbers required for cell-free replication. We also used a comparatively high number of C6/36 cells to have confluent growth and thereby increase the likelihood of *Wolbachia* coming into contact with a C6/36 cell. When *Wolbachia* were killed by heating prior to infection of uninfected C6/36 cells, only minimal amounts of DNA were detected in the cells 6 days post-infection, confirming the results from Nevalainen et al. (2023) that *Wolbachia* are not only passively taken up but also facilitate their uptake. No *Wolbachia* could be detected by immunofluorescence microscopy (data not shown). Thus, detected DNA most probably represents residual DNA from dead *Wolbachia*.

*Wolbachia* growth in the insect cell-free culture was dependent on the initial *Wolbachia* concentrations, with higher concentrations resulting in lower levels of replication. A first explanation might be an insufficient supply of nutrients, e.g., pyruvate and intermediates of the tricarboxylic acid cycle derived from amino acids (Foster et al., 2005). However, in the insect cell-free *Wolbachia* culture, essential and nonessential amino acids are provided in excess by the cell culture medium as well as pyruvate and sugars. *Wolbachia* replicate slowly in the culture and competition for nutrients is unlikely. Instead, *Wolbachia* densities might be regulated by a yet unknown, intrinsic, or host cell-derived mechanism. It is striking that cell-free *w*AlbB showed the highest replication rates at an initial concentration of 0.1–1 × 10^3^ 16S rRNA gene copies/μL. In contrast, in cell-free cultures containing higher densities of *Wolbachia* with 10^4^ or 10^5^ 16S rRNA gene copies/μL, *Wolbachia* numbers only slightly increased. This indicates that *Wolbachia* might sense densities and regulate cell division by internal communication patterns. The two-component regulatory system (TCS) is the predominant form of signaling used in a majority of prokaryotes, including bacteria (Beier and Gross, 2006). It is composed of a sensor histidine kinase and a paired response regulator (Mitrophanov and Groisman, 2008; Jung et al., 2012). Stimuli such as nutrients, osmolarity, oxygen, salinity, and quorum sensing cues are recognized by sensor histidine kinases (Mascher et al., 2006). This activates cognate response regulators which, e.g., coordinate induction of sporulation, regulation of bacterial differentiation, or formation of biofilms (Stock et al., 2000). TCS genes are highly conserved in various *Wolbachia* strains, but very little is known about their function to date (Cheng et al., 2006; Brilli et al., 2010). A bioinformatics study showed that wolbachial TCS genes are consistently found clustered with metabolic genes within several *Wolbachia* strains, including *w*AlbB and *w*Bm (Christensen and Serbus, 2015). Considering these findings, it might be hypothesized that *Wolbachia* are able to sense, e.g., nutrients or quorum sensing molecules and consequently regulate cell division and density. This could explain why cell-free *Wolbachia* growth stops after 9–12 days of incubation and could further explain the observation that *Wolbachia* cell numbers inside C6/36 cells do not reach a density that would negatively affect the survival of their host cell. Nevertheless, how *Wolbachia* growth is regulated remains to be elucidated.

It was also observed that increasing the amount of uninfected C6/36 cells used to prepare total insect cell lysate had a detrimental effect on *Wolbachia* growth rather than increasing replication. *Wolbachia* replication inside their host cells is a complex and tightly regulated process (McGraw et al., 2002; Ruang-areerate et al., 2004). The C6/36 cell culture was originally generated from *A. albopictus* larvae and therefore consists of cells of different cell cycle stages and of different cell types (Singh, 1967; Igarashi, 1978). Hence, it should be considered that *Wolbachia* growth-inhibiting factors present in a subset of C6/36 cells might accumulate when larger numbers of cells are used for lysate preparation. Fraction 1 (containing microsomes and plasma membranes) induced *Wolbachia* growth. However, almost no replication occurred when Fraction 2 (nuclear debris and large organelles) or Fraction 3 (soluble cytoplasmic content) of the C6/36 cells were used to supplement the medium. Since a combination of Fraction 1 with either Fraction 2 or Fraction 3 decreased growth, we hypothesize an inhibiting effect of these fractions. Supplementation with fresh Fraction 1 on day 9 enhanced growth to day 12 but could not extend growth. A second supplementation with fresh Fraction 1 on day 12 also failed to prolong growth. The factor limiting cell-free growth to 12 days remains unclear. As the fresh Fraction 1 was only added to existing cultures and the medium was not completely exchanged, there might be degradation products present that prevented further growth of the cell-free *Wolbachia*.

Notably, it has been shown that survival of endobacteria of the species *Ehrlichia chaffeensis* and *Anaplasma phagocytophilum*, which are closely related to *Wolbachia* spp., is dependent on the incorporation of cholesterol derived from their host cell (Lin and Rikihisa, 2003). Like *Wolbachia*, *Ehrlichia chaffeensis* and *Anaplasma phagocytophilum* do not synthesize lipid A and it was proposed that cholesterol might be necessary to promote membrane stability as a substitute for lipopolysaccharides (Lin and Rikihisa, 2003; Wu et al., 2004). There are indications that *Wolbachia*-infected insect cells might indeed incorporate cholesterol (Caragata et al., 2013; Geoghegan et al., 2017). Further, *Wolbachia* reside in cholesterol-rich Golgi-related vesicles derived from the host which form a vacuole surrounding each bacterium (Cho et al., 2011). Insects assimilate cholesterol from their environment which is incorporated into the plasma membrane and internal membranes such as those from the Golgi apparatus (Rolls et al., 1997). Thus, cholesterol might be a limiting factor for cell-free *w*AlbB replication, and supplementation with the membrane fraction of an insect cell lysate might not be sufficient to sustain growth for more than 12 days. However, the supplementation of water-soluble cholesterol did not lead to increased cell numbers under the conditions tested, indicating that this compound cannot be the only potential limiting growth factor. Apart from cholesterol, eukaryotic sphingomyelin was found in membranes of *Chlamydia trachomatis* (Carabeo et al., 2003), and it was shown that *Chlamydia* need these host lipids for expansion and replication (Feldkamp et al., 2017). Insects do not have sphingomyelin but instead contain ceramide phosphorylethanolamine (Luukkonen et al., 1973). Therefore, the sphingolipids sphingomyelin or ceramide phosphorylethanolamine, respectively, might be taken up by *Wolbachia* residing in different hosts and be essential for replication.

Nevertheless, components of Fraction 1 such as cholesterol cannot be the only necessary factor for *Wolbachia* growth outside their host cell since *Wolbachia* were not able to grow in cell-free medium supplemented with Fraction 1 derived from C6/36 cells harvested in medium without FBS. The composition of FBS is unknown but it is very likely that the serum, similar to the eukaryotic host cells in cell culture, provides proteins, carbohydrates, lipids, vitamins, and other factors essential for *Wolbachia* viability and replication. Similarly, extracellular growth of *Coxiella burnetii* was initially found to be FBS-dependent as well (Omsland et al., 2009), although a defined medium without FBS was developed later (Sandoz et al., 2016).

A prerequisite for bacterial cell division is the proper assembly of the divisome and disturbance of this process results in an aberrant phenotype characterized by swelling or filamentation of bacteria (Goehring et al., 2005; Park et al., 2005). For *Wolbachia* cultured in C6/36 cells, enlarged cells were observed subsequent to the blockade of lipid II biosynthesis by fosfomycin, demonstrating that the cell wall precursor lipid II is essential for the cell division of *Wolbachia* (Vollmer et al., 2013). A similar phenotype was induced by fosfomycin in intracellular *Protochlamydia* and *Waddlia chondrophila* (Pilhofer et al., 2013; Scherler et al., 2020). In the cell-free *Wolbachia* culture, the same aberrant phenotype was observed, indicating that the bacteria are indeed replicating in the cell-free system and that replication can be inhibited by fosfomycin. This was underlined by the fact that we only detected enlarged *Wolbachia* when we measured an increase of 16S rRNA gene copies via qPCR. The fosfomycin-treated cell-free *Wolbachia* were significantly enlarged with 3.36 μm (2.54–4.49 μm) [median (IQR)]. The determined cell diameter of 0.94 μm (0.78–1.36 μm) of the untreated cell-free *Wolbachia* fits well with the 0.8–1.5 μm determined by Hertig, showing that the cell-free *Wolbachia* display their normal morphology (Hertig, 1936).

We hypothesized a phenotype similar to the fosfomycin-induced for other cell wall biosynthesis-inhibiting antibiotics and thus tested ampicillin, bacitracin, and vancomycin. Belonging to the class of beta-lactam antibiotics, ampicillin binds to penicillin-binding proteins (Suginaka et al., 1972; Tipper, 1979). Since bacitracin and vancomycin are large antibiotics that might not be taken up by the C6/36 cells, we were interested in a possible effect on cell-free *Wolbachia*. Bacitracin binds to the pyrophosphate moiety of undecaprenyl pyrophosphate (C55-PP) and vancomycin binds to the d-Ala-d-Ala of lipid II (Perkins, 1969; Storm and Strominger, 1973). For all three, no effect on the phenotype of cell-free *Wolbachia* was observed although their intracellular targets are present (Henrichfreise et al., 2009; Vollmer et al., 2013; Atwal et al., 2021). Possibly, bacitracin and vancomycin are not reaching their targets due to the outer membrane of *Wolbachia* (Nikaido, 1989). Beta-lactams have previously been found to not affect intracellular *Wolbachia w*AlbB in cell culture, the reason is unclear (Fenollar et al., 2003b; Fallon, 2018). In contrast, for *Chlamydia*, an aberrant phenotype is induced by beta-lactams (Matsumoto and Manire, 1970; Kramer and Gordon, 1971), and for *Waddlia chondrophila*, an aberrant phenotype is induced by both beta-lactams and vancomycin (Scherler et al., 2020). Further investigation is necessary to determine why these cell wall biosynthesis-inhibiting antibiotics do not have a similar effect for *Wolbachia*.

Although close attention was paid to using consistent conditions, the cell-free cultures often did not grow. We also observed a decrease in cell-free growth rates over time, which could be due to a new FBS batch (Liu et al., 2023). In the calculation of the median growth rate, only the assays in which the *Wolbachia* replicated were included. The variance of growth rates between independent experiments in cell-free culture containing Fraction 1 was similar to those of *Wolbachia* cultured inside C6/36 cells. In both culture systems, we observed growth variability occurring over time that might originate from variances of medium or cell culture passage. However, the median growth rate of *Wolbachia* in cell-free medium is ~3.5 times lower compared to *Wolbachia* cultured in C6/36 cells. This indicates that in addition to the need for Fraction 1 for cell-free *Wolbachia* cultivation, further constituents are needed.

Previous studies indicate that replication of *Wolbachia* is dependent on the stage of the host life cycle, tissue-specific control mechanism, and host cell replication (Min and Benzer, 1997; McGraw et al., 2002; Ruang-areerate et al., 2004; Landmann et al., 2012). These findings provide insight into the complexity of *Wolbachia* replication, which will in turn influence the cell-free cultivation of the bacteria. In the cell-free culture, the differences in cell types and cell cycle stages of the C6/36 cells used to generate the lysate, and thus Fraction 1, could therefore have a major effect on replication. Further elucidation of this culture system will be necessary to achieve greater and sustained *Wolbachia* growth outside their host cells and to gain insight into the multiple mechanisms that influence and regulate replication in the symbiosis.

Nevertheless, the establishment of this culture system represents a further step in the effort to cultivate *Wolbachia* extracellularly and might also provide important cues for the extracellular cultivation of other endobacteria that could not be cultivated *in vitro* yet. Moreover, a powerful tool for the exploration of *Wolbachia* biology and *Wolbachia*-host interactions is provided by *Wolbachia* cultivated in an insect cell-free *in vitro* system.

## Data availability statement

The raw data supporting the conclusions of this article will be made available by the authors, without undue reservation.

## Author contributions

LB: Data curation, Formal analysis, Funding acquisition, Visualization, Writing – original draft, Writing – review & editing, Investigation, Methodology. KM: Data curation, Formal analysis, Visualization, Writing – original draft, Writing – review & editing, Investigation, Methodology. JV: Data curation, Formal analysis, Visualization, Writing – original draft, Writing – review & editing, Funding acquisition, Investigation, Methodology. CC: Data curation, Formal analysis, Writing – review & editing, Investigation. AS: Conceptualization, Writing – review & editing. AH: Conceptualization, Funding acquisition, Writing – review & editing, Supervision. KP: Conceptualization, Funding acquisition, Project administration, Writing – review & editing, Methodology, Supervision.
