# Supplementary material for: In vitro extracellular replication of Wolbachia endobacteria
Source: Front Microbiol. 2024 Jul 18;15:1405287. doi: 10.3389/fmicb.2024.1405287 (PMC11293327; doi:10.3389/fmicb.2024.1405287)
Supplement: Supplementary file 1 [file Data_Sheet_1.pdf]

Supplementary Material

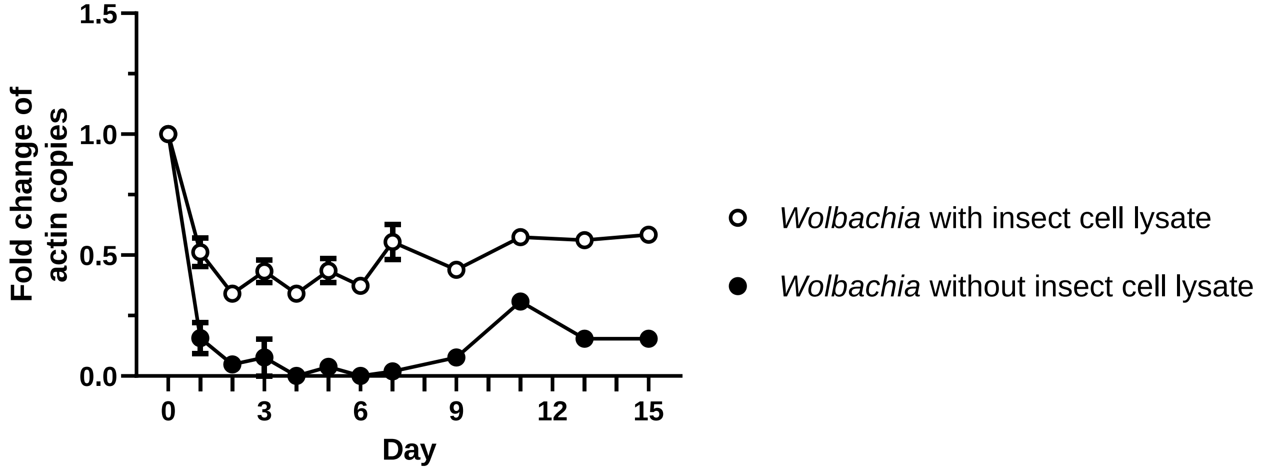

Supplementary Figure 1. Cell lysate is free of C6/36 cells. *Wolbachia* were purified from C6/36 cells and incubated in medium as described (Rasgon et al., 2006), or were purified by an abbreviated protocol that retained the insect cell lysate. Cell-free cultures were incubated at 26 °C for 15 days and samples were taken every one to three days. Actin copy numbers were quantified by qPCR. Copy numbers were normalized to day 0. Data were pooled from two independent experiments. For days 2, 4, 6 (experiment 1) and days 9, 11, 15 (experiment 2), the data from only one experiment is shown. For the other days, the mean ± SEM of 2–3 wells is shown.

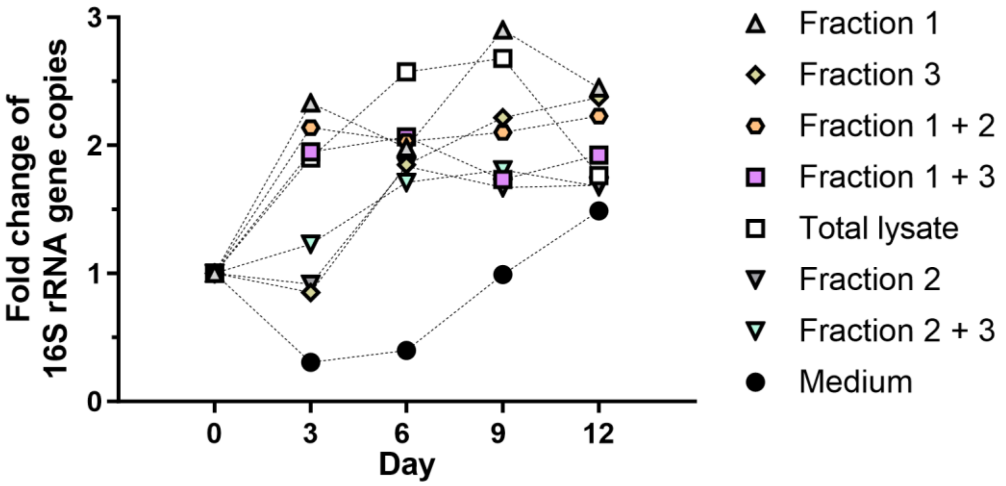

Supplementary Figure 2. Supplementation with Fraction 1 supports wolbachial growth more than any other fraction or combination of fractions. Cell-free *Wolbachia* (0.5 x 10^3 16S rRNA gene copies/µL) were incubated with Fraction 1, Fraction 2, or Fraction 3 or combinations of these fractions from uninfected C6/36 cells (equivalent to 0.95 x 10^6 cells/mL) at 26 °C for 12 days. Growth was monitored by 16S rRNA gene qPCR every three days and data were normalized to day 0. For every time point, the mean of five wells is shown.

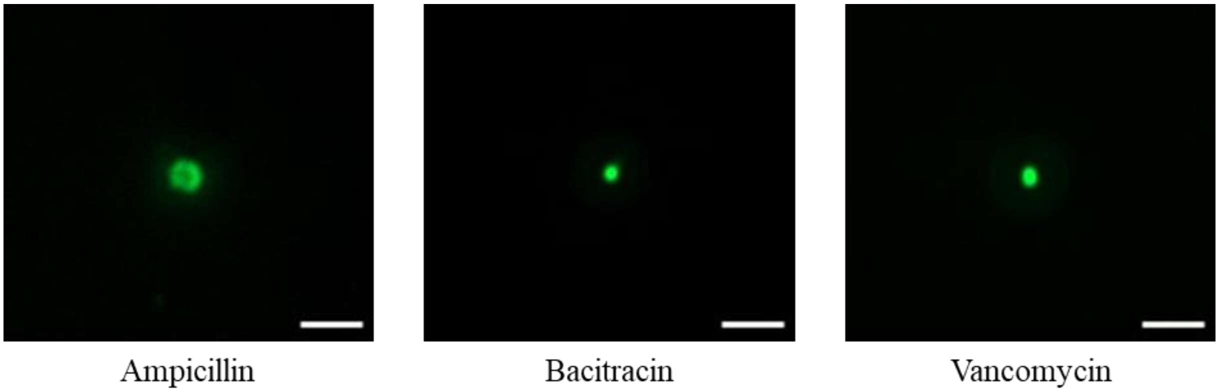

Supplementary Figure 3. Cell-free *Wolbachia* morphology after treatment with cell wall biosynthesis-inhibiting antibiotics. Cell-free *Wolbachia* (0.5 x 10^3 16S rRNA gene copies/µL) were incubated with Fraction 1 from uninfected C6/36 cells (equivalent to 0.95 x 10^6 cells/mL) at 26 °C for 12 days and treated with 512 µg/mL ampicillin, bacitracin, or vancomycin every three days. Cells were fixed and visualized by immunofluorescence microscopy using *w*PAL anti-serum and an Alexa 488-conjugated secondary antibody. Scale bar: 2 µm.
